# A Cell-Based Assessment of the Muscle Anabolic Potential of Blue Whiting (*Micromesistius poutassou*) Protein Hydrolysates

**DOI:** 10.3390/ijms24032001

**Published:** 2023-01-19

**Authors:** Niloofar Shekoohi, Miryam Amigo-Benavent, Guilherme Wesley Peixoto da Fonseca, Pádraigín A. Harnedy-Rothwell, Richard J. FitzGerald, Brian P. Carson

**Affiliations:** 1 Department of Biological Sciences, University of Limerick, V94 T9PX Limerick, Ireland; 2 Health Research Institute, University of Limerick, V94 T9PX Limerick, Ireland; 3 Heart Institute (InCor), University of São Paulo Medical School, São Paulo 01246-903, Brazil; 4 Department of Physical Education and Sport Sciences, Faculty of Education and Health Sciences, University of Limerick, V94 T9PX Limerick, Ireland

**Keywords:** blue whiting protein hydrolysates, C2C12 cells, muscle growth, muscle protein synthesis

## Abstract

Blue whiting (BW) represents an underutilised fish species containing a high-quality protein and amino acid (AA) profile with numerous potentially bioactive peptide sequences, making BW an economic and sustainable alternative source of protein. This study investigated the impact of three different BW protein hydrolysates (BWPH-X, Y and Z) on growth, proliferation and muscle protein synthesis (MPS) in skeletal muscle (C2C12) myotubes. BWPHs were hydrolysed using different enzymatic and heat exposures and underwent simulated gastrointestinal digestion (SGID), each resulting in a high degree of hydrolysis (33.41–37.29%) and high quantities of low molecular mass peptides (86.17–97.12% <1 kDa). C2C12 myotubes were treated with 1 mg protein equivalent/mL of SGID-BWPHs for 4 h. Muscle growth and myotube thickness were analysed using an xCelligence™ platform. Anabolic signalling (phosphorylation of mTOR, rpS6 and 4E-BP1) and MPS measured by puromycin incorporation were assessed using immunoblotting. BWPH-X significantly increased muscle growth (*p* < 0.01) and myotube thickness (*p* < 0.0001) compared to the negative control (amino acid and serum free media). Muscle protein synthesis (MPS), as measured by puromycin incorporation, was significantly higher after incubation with BWPH-X compared with the negative control, but did not significantly change in response to BWPH-Y and Z treatments. Taken together, these preliminary findings demonstrate the anabolic potential of some but not all BWPHs on muscle enhancement, thus providing justification for human dietary intervention studies to confirm and translate the results of such investigations to dietary recommendations and practices.

## 1. Introduction

Muscle mass, strength and function are progressively lost with aging [1]. A loss or reduction in skeletal muscle function often leads to increased morbidity and mortality either directly, or indirectly, via the development of secondary diseases such as diabetes, obesity and cardiovascular disease [1,2]. Maintaining skeletal muscle function throughout the lifespan into old age is essential for independent living and good health. The efficient operation of the processes that regulate muscle development, growth, regeneration and metabolism is required for skeletal muscle to function at optimal levels [3]. Older adults are more prone to limited physical activity and periods of bed rest compared to younger persons and consequently, they are prone to loosing lean tissue/muscle mass [4]. Muscular atrophy in the elderly principally occurs due to a diminished ability to synthesise muscle protein [5]. This could be due to anabolic blunting, characterised by decreased anabolic signalling in response to aminoacidemia in the elderly [6,7], along with lower levels of protein consumption in older adults compared to their younger counterparts [8].

High-quality dietary proteins, containing all essential amino acids (EAA), are a critical macronutrient for enhancing muscle and general metabolic health [9]. The muscle health benefits of dietary protein intake are of interest to different population groups, e.g., those engaged in sports and the elderly. Protein is composed of amino acids that can induce a muscle protein anabolic response by the supply of EAA, particularly branched-chain amino acids (BCAAs) such as leucine, isoleucine and valine [10]. Low intakes of BCAAs are associated with reduced muscle mass, poor muscle function and reduced muscle strength in community-dwelling older individuals [11]. Acute postprandial aminoacidemia, which occurs after consuming dietary protein, is a key driver of muscle protein synthesis (MPS), the amplitude and duration of which is controlled substantially by the quality of the protein ingested [12,13,14]. There are several signalling pathways through which proteins, peptides and amino acids drive MPS [15]. Leucine, BCAAs and essential amino acids (EAA) are particularly potent activators of these pathways [16,17] leading to a net increase in MPS over muscle protein breakdown (MPB) [18]. Thus, identifying the molecular mechanisms that regulate protein degradation and particularly protein synthesis is critical for the development of potential interventions that can protect muscle mass and function.

A recent detailed review examined the anabolic potential of protein hydrolysates compared to intact proteins, exploring and synthesising evidence on the purported mechanistic benefits such as more rapid AA delivery, higher proportion of di-, tri- and smaller oligo-peptides reaching the circulation and subsequent potential to promote skeletal muscle protein remodelling [19]. Fish represents a rich source of high-quality protein [20,21,22]. Protein hydrolysates/peptides derived from fish and fish processing co-products by-products have potential applications as high-value functional food ingredients in the maintenance of muscle mass and health [23,24]. Therefore, valorisation of fish protein for these purposes (MPS) may lead to an enhancement in their commercial value and their environmental efficiency/sustainability [25,26]. It has been reported that humans may benefit from the ingestion of fish-derived protein hydrolysates (FPH) to support healthy aging, metabolic health and skeletal muscle metabolism [27]. Cordeiro et al. (2020) showed immediate and robust post-exercise aminoacidemia in young people after consuming 0.25 g/kg body mass of Nile tilapia-derived FPH. This demonstrated that FPH ingestion may be a viable and effective option for increasing skeletal muscle anabolism [28]. With a protein content of approximately 18.6% (*w*/*w*), blue whiting (*Micromesistius poutassou*)*,* an underutilised fish species, represents one such marine protein source [29]. Enzymatic hydrolysates of blue whiting proteins have shown health enhancing properties both in vitro and in vivo [30,31,32]. Lees et al. (2021) previously showed that a BW protein hydrolysate (BWPH) induced robust essential aminoacidemia in older adults and also provided preliminary evidence of the effects of BWPH treatment on MPS and myotube growth in skeletal muscle (C2C12) cells [24].

An understanding of if and how different proteins may improve MPS is critical for selecting appropriate dietary protein sources for people of all ages, but possibly even more so for those who are experiencing or are at risk of muscle wasting, such as the elderly or the hospitalised. BWPHs were subjected to in vitro simulated gastrointestinal digestion (SGID) prior to exposure to a muscle cell (C2C12) grown in culture to simulate the digestion conditions in the human body, and therefore to investigate the role of enzymatic breakdown and other modulations in the GI tract on the responses given by the peptides. This study employed a screening assay to evaluate the anabolic potential of three SGID-treated BWPHs on the growth, proliferation, signalling and de novo MPS in C2C12 skeletal muscle myotubes. It was hypothesised that myotubes incubated in medium conditioned with SGID treated BWPHs would display increased growth, myotube diameter, intracellular signalling and MPS compared to serum and amino acid free media that acted as a negative control.

## 2. Results

### 2.1. Degree of Hydrolysis (DH) and Molecular Mass Distribution

The BWPHs (BWPH-X, -Y and -Z) were subjected to SGID in vitro in order to mimic in vivo conditions. As shown in Table 1, the degree of hydrolysis (DH) increased significantly (*p* < 0.05) following SGID of all BWPHs (33.41 ± 0.91–37.29 ± 0.24%). The SGID-BWPHs showing high DHs contained lower quantities of high molecular weight components and higher quantities of components < 1 kDa (Table 1). This would indicate that all BWPH’s were further degraded during SGID.

### 2.2. Total Amino Acid Analysis

The amino acid composition of the BWPHs is shown in Table 2. The BWPHs contain all essential amino acids (EAAs), with Leu accounting for 53.7–56.2 g·kg^−^^1^ of the total amino acids. The most abundant amino acid found in the hydrolysates was Glx which represented 133.7–146.0 g·kg^−^^1^ of the total amino acids. Asx, Lys, Gly, Arg and Ala were found in relatively high abundance for all hydrolysates. The BCAA (Ile, Leu, Val) content was 109.2–114.9 g·kg^−^^1^.

### 2.3. Cell Viability Assay (WST-1)

**Figure 1:**
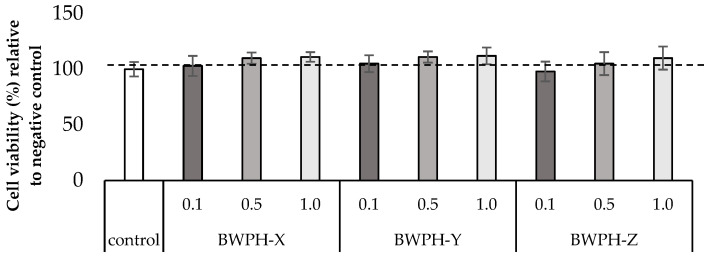
Viability of muscle myotube (C2C12) cells treated with 0.1, 0.5 and 1.0 mg·mL^−1^ protein equivalent blue whiting (*Micromesistius poutassou*) protein hydrolysates (BWPHs). The negative control was amino acid and serum free media without BWPHs. Treatment of cells was for 4 h after 1 h of nutrient deprivation. The results represent mean ± SD (n = 3) and are expressed relative to the negative control.

Differentiated myotubes were nutrient deprived for 1 h before carrying out cell viability assays for 4 h using amino acid and serum free media as a control with 0.1, 0.5 and 1.0 mg·mL^−1^ protein equivalent of the SGID-BWPHs. Cell viability, as measured using the WST-1 assay, was between 98–112% after 4 h incubation with different concentrations of the SGID-BWPHs relative to the control condition (Figure 1). Treatment of cells with up to 1 mg·mL^−1^ protein equivalent SGID-BWPHs for 4 h, after 1 h of nutrient deprivation, led to no negative effect on cell viability at all time points compared with the control condition. As there was no reduction in cell viability, a BWPH concentration of 1 mg·mL^−1^ protein equivalent was used for subsequent treatments.

### 2.4. Myotube Growth and Thickness

**Figure 2:**
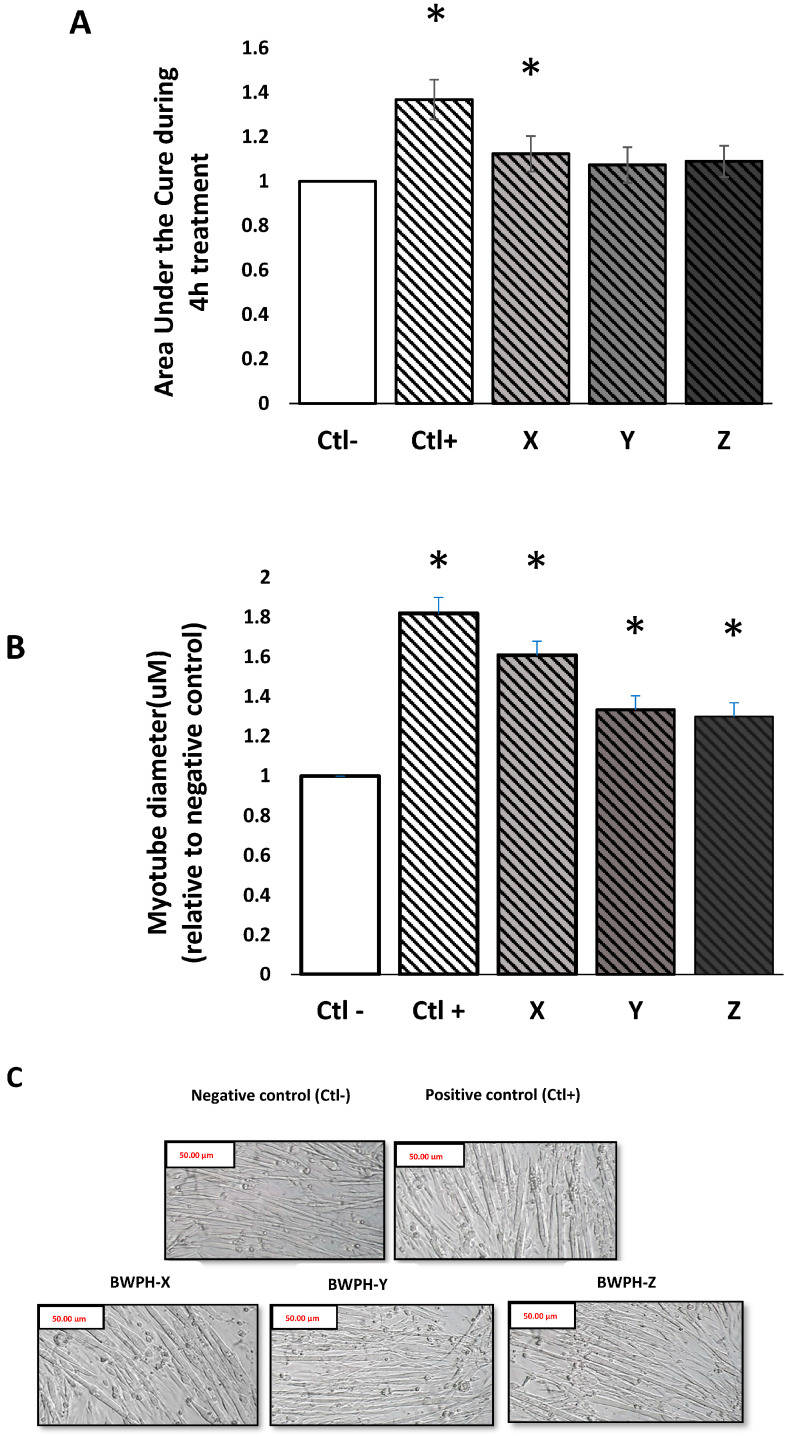
Effect of blue whiting (*Micromesistius poutassou*) protein hydrolysate (BWPH) treatment on AUC and myotube diameter in skeletal muscle cells. C2C12 myotubes were nutrient deprived for 1 h followed by 4 h treatment with 1 mg·mL^−1^ protein equivalent of BWPH-X, Y and Z. Myotube growth was monitored every 2 min over 4 h. (**A**) Representative graph comparing myotube growth (AUC) in the presence of different samples relative to the negative control. (**B**) Representative graph comparing myotube diameter in the presence of different samples relative to the negative control (**C**) Quantification of myotube diameter taken 4 h post treatment as measured by microscopy. Images of myotubes treated with samples were taken at 4× magnification following 4 h treatment. All values are expressed as mean ± SD (n = 6) for 3 plates (duplicate in each plate). *p* < 0.05 * compared to the negative control (amino acid and serum free media). Ctl−: negative control (amino acid and serum free media), Ctl+: positive control (100 ng·mL^−1^ IGF-1), X: SGID-BWPH-X, Y: SGID-BWPH-Y, Z: SGID-BWPH-Z.

The area under the curve (AUC) of the cell index (CI) was significantly (*p* < 0.05) increased in response to BWPH-X treatment compared to the negative control, but did not change in response to treatment with the other BWPHs (Figure 2A). The IGF-1 (positive control) CI values were also significantly higher (*p* < 0.05) in comparison to the negative control during the treatment period which was as expected. In order to validate changes in myotube diameter based on these results, myotube thickness was measured following 4 h treatment using ImageJ™ software. Myotube diameter was significantly increased with all three SGID-BWPHs treatment compared to the negative control (*p* < 0.001) (Figure 2B).

### 2.5. Muscle Protein Synthesis

Activation of the mammalian target of rapamycin (mTOR) in response to the BWPHs treatment was reported as the ratio of phosphoproteins relative to the total protein. All values were then expressed as a percent of the negative control within each assay.

**Figure 3:**
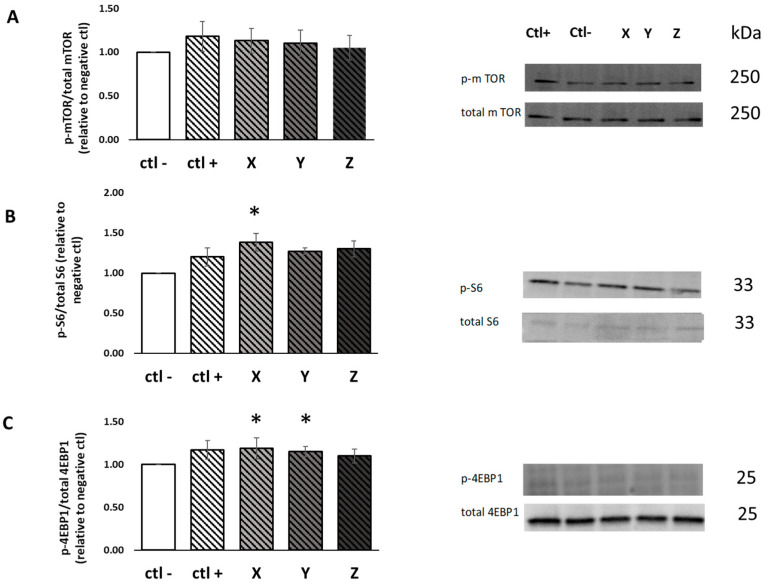

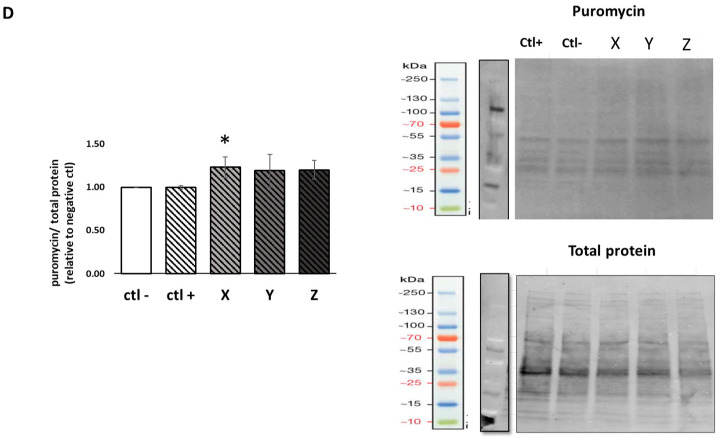
Phosphorylation of mTOR, 4EBP1 and ribosomal S6 incubated with 1 mg·mL^−1^ protein equivalent BWPH-X, Y and Z (n = 4). C2C12 myotubes were nutrient deprived for 1 h followed by treatment with SGID-BWPHs plus 1 µM puromycin for 4 h. Data reported as the ratio of phosphoproteins relative to the total protein. All values were expressed as a percent of the negative control within each assay. Phosphorylation of mTOR (**A**), rpS6 (**B**) and 4EB-P1 (**C**) following SGID-BWPH treatment and their corresponding representative immunoblot. (**D**) Muscle protein synthesis (MPS) after treatment with SGID-BWPHs and their representative immunoblot of MPS (measured by puromycin incorporation) relative to total protein (loading control). Data reported as mean ± SEM, * compared to negative control, *p* < 0.01. Ctl−: negative control (amino acid and serum free media), Ctl+: positive control (100 ng·mL^−1^ IGF-1), X: SGID-BWPH-X, Y: SGID-BWPH-Y, Z: SGID-BWPH-Z.

The expression of mTOR phosphorylation was numerically higher with all three SGID-BWPHs; however, this increase was not significantly different to the negative control (*p* > 0.05) (Figure 3A). rpS6 phosphorylation was significantly increased in response to BWPH-X compared to the negative control (1.38-fold increase, *p* < 0.05), but did not significantly change in response to other BWPH-treatments (Figure 3B). Stimulation of the downstream targets of mTOR activation, 4E-BP1, was significantly higher in SGID-BWPH-X and Y compared to the negative control (1.15- and 1.17-fold increase, *p* < 0.05) (Figure 3C).

The SunSET technique was employed to quantify MPS and to verify whether mTOR, rpS6 and 4E-BP1 activation caused an increase in MPS in skeletal muscle cells (C2C12 myotubes) following 4 h treatment. The results obtained herein showed that puromycin incorporation was significantly increased in response to BWPH-X treatment compared to the negative control (1.23-fold increase, *p* < 0.05), but did not significantly change in response to other BWPHs treatment (Figure 3D). These results demonstrate that SGID-BWPH-X may have the potential to promote MPS mediated by signalling through the mTOR pathway in skeletal muscle cells.

## 3. Discussion

BW, a low-value underutilised fish species, contains high-value nutritional and health enhancing components. BW-derived proteins, including protein hydrolysates, have previously been shown to stimulate essential aminoacidemia and MPS in vitro and in vivo [24]; therefore, they may be considered as potential high quality dietary sources of protein for the prevention of muscle loss and function.

To our knowledge, this study is the first to screen and compare the anabolic potential of several BWPHs in order to establish those with the greatest potential to enhance myotube growth, proliferation and MPS. In order to better represent the in vivo processing of BWPHs and thus enhance the potential translation of these findings, hydrolysates were subjected to SGID prior to presentation to C2C12 cells.

The DH and molecular mass distribution data showed that all samples were further digested during SGID (Table 2). The molecular mass distribution profile was influenced by the degree of hydrolysis, as the proportion of peptides < 1 kDa increased in all samples following hydrolysis and SGID. Total amino acid profiling of BWPHs revealed high levels of known regulators of MPS including BCAA and EAA, particularly leucine, which may have contributed to the anabolic potential observed particularly with BWPH-X. The minor differences in AA profiles of these three samples could be related to the differences in moisture, salt content and different hydrolysis conditions (enzymes, pH, temperature). In agreement with the results presented here, Lees et al. [24] previously showed that a BWPH generated under different conditions possessed a high concentration of these amino acids, and consumption of BWPH induced postprandial essential aminoacidemia in older adults, sufficient to stimulate MPS in vitro.

C2C12 myotube cells were treated with BWPHs, using a previously published experimental model [33,34] to investigate the potential of BWPHs to stimulate myotube growth and activate MPS. Skeletal muscle mass is regulated through several integrated signalling pathways. IGF-1 is a well-known regulator of muscle mass and hypertrophy [35]. IGF-1 is one of the best-characterised growth factors, and it has been shown to modulate muscle size and play a critical role in regulating muscle function [36]. Thus, 100 ng·mL^−1^ IGF-1 [37] was used as a positive control to determine the positive stimulation achievable in the assay. Based on the data obtained from the xCELLigence™ platform, SGID-BWPH-Y and Z showed no significant difference in AUC during the treatment time compared to the negative control (Figure 2A). However, a significant increase for BWPH-X was observed based on AUC analysis (Figure 2A) which indicates that this sample may have the potential to increase myotube growth, which may lead to stimulation of MPS. In order to validate the experimental approach regarding the effect of the SGID-BWPHs on myotube growth, images of myotubes post addition of SGID-BWPHs and control treatments were taken. All SGID-BWPHs significantly increased myotube diameter (Figure 2B), indicating that these samples may boost muscle hypertrophy. These results support the findings of Lees et al. (2021) who previously showed that C2C12 cells demonstrated greater hypertrophy with BPWH-fed serum of healthy older adults following 4 h treatment, compared with non-essential amino acid fed serum based on myotube thickness (*p* = 0.028; d = 1.24) [24].

mTOR and its downstream pathway are key regulators of MPS and by extension, muscle hypertrophy. The ability of the SGID-BWPHs treatments to stimulate mTOR signalling was assessed by measuring the phosphorylation of mTOR and its downstream signalling molecules 4EBP-1 and rpS6. Many studies have identified this critical signalling molecule as a key mediator in translating activation of mTOR to activation of muscle protein MPS [38,39,40,41]. In this study, the activation of mTOR and its downstream kinases, rpS6 and 4E-BP1, following cell treatment with the SGID-BWPHs and related controls was measured. No significant increase in mTOR signalling was observed; however, an increase in signalling of 4E-BP-1, in response to both BWPH X and Y, was seen herein. Although 4E-BP1 regulation and its function (as targets for mTOR signalling) in the control of mRNA translation have received a lot of attention, their significance for the regulation of total protein synthesis is yet unknown. Furthermore, the activation of rpS6K, which can take part in binding mRNA and have a regulatory role in translation initiation, was measured. A significantly increased stimulation of rps6K by BWPH-X was observed. Taken together, it appears that no condition was sufficient to activate mTOR to a greater extent than the negative control, or that perhaps this marker was not sufficiently sensitive to detect differences between conditions. This is supported by the fact that there were different responses to some SGID BWPHs in downstream markers of both protein elongation and translation. Previous work from Lees et al. (2021) [24] observed a similar response for both mTOR and downstream markers in response to treatment with both fish and milk protein sources. This suggests that these downstream markers, which indicate increased activation of protein translation, may be more sensitive to subtle anabolic differences. This also suggests that these BWPHs have the ability to increase MPS. This was tested by using the SUnSET technique [42]; whereby, puromycin incorporation was used as a proxy for de novo MPS. The time course of treatment with puromycin to see a detectable response had been validated previously [33]. Based on previous findings, puromycin incorporation was greater when included for the entire time of the treatment (up to 4 h) [33]. BWPH-X again showed a significant increase in MPS compared to the negative control. This supports the findings of the signalling data and corroborates the data from the cell index and myotube diameter data. Taken together, these data suggest that BWPH-X has the potential to stimulate MPS.

To the best of our knowledge, there is just one other study which to date reports on assessment of the direct effect of fish protein sources on C2C12 cells. Roeseler et al. (2017) developed a screening assay employing C2C12 murine muscle cells to assess the relative abilities of several commercial protein sources and experimental soy protein hydrolysates, following simulated gut digestion (SGD), to activate the mTORC1 MPS signalling pathway (p70S6K(Thr389) phosphorylation). They reported higher bioactivity with fish-derived proteins compared to other protein sources; however, it was not significantly different from other protein sources. However, there were some limitations in the study because it primarily focused on the phosphorylation of p70S6K, even though there are other signalling molecules that may be variably affected by different proteins (e.g., 4EBP-1 and rpS6). Furthermore, they did not measure protein synthesis through MPS measurement methods; therefore, it is not known whether the increase in mTORC1 signalling may translate into increased protein synthesis [43].

In agreement with the results herein, there are also some animal studies indicating the beneficial effects of marine protein hydrolysates on muscle hypertrophy and MPS via the mTOR pathway. Jeon et al. (2021) assessed the effects of oyster hydrolysates as a high-quality marine protein source on muscle atrophy in both C2C12 cell and in mice. In vitro, the hydrolysates recovered the dexamethasone-induced reduction in myotube diameter. In vivo, the hydrolysates not only improved grip strength and exercise endurance, but also attenuated the loss of muscle mass. Mechanistically, the hydrolysates increased the expression of protein synthesis-related protein levels via phosphoinositide 3-kinase (PI3K)/protein kinase B (Akt)/mammalian target of the rapamycin pathway [44]. Furthermore, two animal studies exist which assessed the effects of dietary *Alaska pollack* protein (APP) intake on the induction of acute and sustainable skeletal muscle hypertrophy in male 5-week-old Sprague–Dawley rats [45,46]. It was found that dietary APP significantly increased gastrocnemius muscle mass (110%) after 56 days of feeding. However, the puromycin-labelled peptides were not different between dietary casein and APP after 2 days of feeding. According to these findings, dietary APP does not stimulate acute protein synthesis in the gastrocnemius muscle. Furthermore, skeletal muscle hypertrophy arising from APP intake occurs over a few days [45]. In keeping with these studies, Morisasa et al. (2022) indicated that APP promoted skeletal muscle hypertrophy by activating the Akt/mTOR signalling pathway [46].

In conclusion, the data presented herein indicates that some BWPHs, as a high-quality protein source, can have a significant impact on muscle growth and MPS. BWPH-X stimulated MPS as measured by myotube growth, size, mTOR signalling and puromycin incorporation in C2C12 myotubes. It is possible that the activity seen may be the result of various peptides and amino acids acting on their own or, more likely, functioning additively or even synergistically. A recent review by Morgan and Breen outlined the potential for bioactive peptides from protein hydrolysates, but concluded that more research is required in this space at both the basic science and human intervention levels [19]. Further research is needed to determine the peptides/amino acids that may be responsible for the observed activity through bioassay-driven fractionation of the hydrolysates, and it is necessary to carry out confirmatory studies using the identified synthetic peptides/amino acids or combinations of those for anabolic potential in skeletal muscle. Ultimately, human dietary intervention studies will be essential to confirm and translate the results of such investigations to dietary recommendations and practices.

## 4. Materials and Methods

### 4.1. Chemicals and Reagents

Hydrochloric acid (HCl) was from VWR (Dublin, Ireland). Sodium hydroxide (NaOH) and acetic acid were from Fisher Scientific (Dublin, Ireland). Boric acid, Kjeldahl catalyst tablets (free of Hg and Se) and sulphuric acid ≥ 98% were from Sigma Chemical Company (Dublin, Ireland). The enzyme preparations, BC pepsin (P6887, ≥3200 units/mg protein) and Pancreatin (P7545, 4 × USP specifications) were both purchased from Sigma-Aldrich (Dublin, Ireland). Trinitrobenzene sulphonic acid (TNBS) reagent was from Medical Supply Co Ltd. (Dublin, Ireland). All other reagents were of analytical grade unless stated otherwise.

The C2C12 (subclone C2/4) mouse adherent myoblast cell line was purchased from ATCC^®^ CRL1772, Manassas, VA (Lot number 60339292). Dulbecco’s Modified Eagles Medium (DMEM) (D6429), Fetal bovine serum (FBS) (F7524), penicillin/streptomycin (P0781), L-glutamine (G7513), horse serum (H1270), D-glucose, cell proliferation reagent WST-1, Tris/HCl pH 7.4 (T1503), sodium orthovanadate (Na_3_VO_4_) (S6508) phenylmethanesulfonylfluoride (PMSF) (P7626), Pepstatin (P4265) and Aprotinin were all purchased from Sigma-Aldrich (Dublin, Ireland). DMEM amino acid and serum-free medium (D9807-10) was from US Biological (Salem, MA, USA). Sodium pyruvate (GE Healthcare, Thermo-Fisher) and Precision Plus Protein™ Dual Colour Standards (SM1811) were both purchased from Thermo-Fisher (Dublin, Ireland). Human insulin growth factor-1 (IGF-1) (100-11) was from PEPROTECH (London, UK). SDS-PAGE precast gels (4–15% Mini-Protean TGX Stain-free; Bio-Rad 456-8083) were obtained from Accuscience (Dublin, Ireland).

All primary antibodies for mTOR, phosphor (p)mTOR, 4-EBP1, p4-EBP1, S6 Ribosomal Protein, pS6 Ribosomal Protein, β-actin (#2972S, #5536S, #9644S, #2855S, #2217S, #5364S, #3700S) were from Cell Signalling (Bioke, Leiden, The Netherlands). Puromycin (MABE343 anti-puromycin, clone 12D10 mouse monoclonal) was purchased from Sigma-Aldrich (Dublin, Ireland). Secondary antibody green rabbit (926- 32211 IRDye 800 CV) and goat anti-rabbit IgG were obtained from LI-COR Biosciences UK Ltd. (Cambridge, UK).

### 4.2. Generation of BWPHs by Direct Enzymatic Hydrolysis

The BWPH samples were produced at industrial scale under GMP conditions from thawed minced blue whiting at BioMarine Ingredients Ireland Ltd. (Lough Egish Food Park, Castleblaney, Co., Monaghan, Ireland). All employed enzyme preparations were food-grade and of microbial origin. After hydrolysis, the enzymes were inactivated at 90 °C for 15 min and the bones were separated using a vibrating sieve. Using a two-step centrifugation process, the water-soluble fraction was separated from the undigested residue. This soluble phase was then ultrafiltrated and dehydrated to approx. 50% (*w*/*w*) total solids, prior to spray drying [30]. Three BWPHs were generated at pilot scale for this study and they are named BWPH-X, Y and Z. BWPH-X is a membrane processed equivalent of BWPH-Y. It was diluted to a greater extent before a nanofiltration step to remove extra salt.

### 4.3. Simulated Gastrointestinal Digestion (SGID)

SGID was carried out as described by Walsh et al. [47] with some modifications. In brief, the hydrolysate (2.0% (*w*/*v*) protein equivalent) was incubated at 37 °C and pH 2.0 for 90 min with pepsin at an E:S of 2.5% (*w*/*w*). The pH was subsequently adjusted to pH 7.0 and the samples were then incubated for a further 150 min at 37 °C with Pancreatin at an E:S of 1% (*w*/*w*), and the reaction was then inactivated by heating at 85 °C for 15 min. All samples were subsequently freeze-dried and were stored at −20 °C until use.

### 4.4. Amino Acid Quantification, Degree of Hydrolysis (DH) and Molecular Mass Distribution Profiles

The protein equivalent content of the samples was determined using the macro-Kjeldahl procedure [48] using a Nitrogen to protein conversion factor of 5.89 [30]. All analyses were performed in triplicate. The TNBS method, as described by Harnedy et al. [23], was used to estimate DH. Total amino acid analysis of the hydrolysates was performed by external contract suppliers.

The DH was calculated following the equation: DH (%) = 100 × ((AN2 − AN1)/Npb). Where, AN1 is the amino nitrogen content of the protein substrate (mg/g protein) which is equal to 5.6 mg amino nitrogen/ g protein, AN2 is to the amino nitrogen content of the hydrolysate (mg/g protein) under investigation and Npb is the nitrogen content of the peptide bonds in the unhydrolyzed protein (102.3 for fish muscle) [49]. Gel permeation-high performance liquid chromatography (GP-HPLC) was used to assess the molecular mass distribution profiles of the BWPH and SGID samples as previously described [50].

### 4.5. Cell Culture

Undifferentiated C2C12 myoblasts were cultured at 37 °C in a humidified atmosphere containing 5% CO_2_ in growth media (DMEM including 10% (*v/v*) FBS, 1% (*v/v*) penicillin/streptomycin and 1% L-glutamine). After the cells had reached 70–80% confluence, differentiation was promoted for up to 6 days using DMEM supplemented with 2% horse serum as previously reported [33]. Over the next six days, media were replaced every 24 h and myotube formation was tracked until full myotube formation was attained. Before treatment with BWPHs, fully differentiated myotubes were deprived of nutrients for 1 h in DMEM amino acid and serum-free medium, which contained 1 mM sodium pyruvate, 1% (*v/v*) penicillin/streptomycin solution, 1 mM L-glutamine, 6 mM D-glucose and 34 mM NaCl (pH adjusted to 7.3). After serum and amino acid starvation, the C2C12 cells were treated with the SGID BWPHs in DMEM amino acid and serum-free medium for 4 h at 1 mg·mL^−1^ protein equivalent final concentration. Cells incubated with DMEM amino acid and serum-free medium alone served as negative control for the assay, while 100 ng·mL^−1^ IGF-1 was used as a positive control.

### 4.6. WST-1 Cell Viability Assay

Cells were seeded and differentiated in 96-well plates in a final volume of 100 μL culture medium/well. Fully differentiated cells were washed with DMEM amino acid and serum free medium and were allowed to incubate with the DMEM amino acid and serum-free medium for 1 h. DMEM amino acid and serum free medium alone (negative control) and different concentrations (1, 0.5, 0.1 mg protein equivalent/mL) of SGID treated hydrolysates were added to each well and incubated for 4 h. The WST-1 Cell viability assay was carried out following the suppliers’ instructions. Viable cells were evaluated using absorbance measurements at 450 nm. The cell viability was normalised with the number of control cells.

### 4.7. Electrical Impedance Measurement

Label free, non-invasive, electric impedance measurements were taken using an xCELLigence^TM^ Real Time Cell Analysis (RTCA) instrument (ACEA Biosciences, Inc., San Diego, CA, USA) using a microelectronic E-16 well gold plated base sensor plate (ACEA Biosciences) [51]. C2C12 myoblasts were seeded at a density of 5000 cells per well in a volume of 0.2 mL on an electrode-coated plate. Cells were allowed to proliferate for 30 h (optimised for 70% confluency) before changing to differentiation media and then differentiation was induced. An automated reading of cell status, represented as cell index (CI), was taken in real time throughout the myoblast proliferation to the myotube formation cycle. CI recordings were taken every 15 min during myoblast proliferation and every 2 min during myotube formation, throughout the incubation with hydrolysates. Data were normalised to the start of the treatment phase of the experiment.

### 4.8. Myotube Diameter Measurement

Myotubes were photographed with a digital camera attached to a microscope (Olympus CKX31, Tokyo, Japan). To quantify changes in myotube thickness, images were stored as JPEG files and analysed using Image J software (National Institutes of Health, Baltimore, MD, USA). For each treatment condition, three diameter measurements were collected along each myotube in at least six separate fields for a total of at least 100 myotubes. The average myotube diameter was used to represent each treatment condition in the analysis.

### 4.9. Preparation of Cellular Protein Lysates

Cellular protein extracts were prepared by placing cells on ice, removing media and rinsing three times in ice cold phosphate buffered saline. Cells were lysed using cold cell lysis buffer (10 mM Tris/HCl pH 7.4, 150 mM NaCl, NaF, 1% Na_4_P_2_O_7_) containing phosphatase inhibitor (Na_3_VO_4_ (1 mM) and protease inhibitors (phenylmethanesulfonylfluoride fluoride (PMSF) (1 mM), pepstatin (1 μM) and aprotinin (1.5 μg/mL)). Cell lysates were incubated on ice for 30 min and then each plate was scraped using a cell scraper; homogenates were centrifuged at 130× *g* for 10 min at 4 °C to remove nuclei and cellular debris. Supernatants were analysed for total protein using the Bradford assay following the suppliers’ instructions (Bio-Rad Protein Assay Dye Reagent, Accuscience Ireland Ltd.) and boiled in sample buffer for sodium dodecyl sulfate–polyacrylamide gel electrophoresis (SDS-PAGE) analysis.

### 4.10. Western Blot Method and Analysis

Protein lysates (30 μg) and ladders Precision Plus Protein^TM^ Dual Colour Standards (5 μL) were loaded on 4–15% linear gradient SDS-PAGE precast gels. After electrophoresis, gels were activated by ultraviolet light for stain-free technology using an UVITEC Cambridge Imaging system (UVITEC; Cambridge, UK) (300 nm UV light for 1 min). The proteins captured in the gel were transferred to a nitrocellulose membrane using the semi-dry transfer technique (Trans-blotR Turbo^TM^; Bio-Rad). Following transfer, membranes were blocked for 1 h at room temperature with 5% (*w/v*) skimmed milk powder in 1× Tris Buffered saline containing 0.5% Tween-20 (TBST). Membranes were incubated overnight at 4 °C with a primary antibody. All primary antibodies were diluted 1:1000 in 5% BSA in TBST: mTOR, phosphor (p)mTOR, P70S6K, pP70S6K, 4-EBP1, p4-EBP1, S6 Ribosomal Protein, pS6 Ribosomal Protein, β-actin and puromycin. After incubation, membranes were washed four times and incubated with the secondary antibody green rabbit for 1 h for all primary antibodies with the exception of puromycin, which was incubated with goat anti-mouse IgG2a-specific. Images were captured using a UVITEC Cambridge Imaging system and whole-lane band densitometry was quantified using NineAlliance UVITEC Software. Following probing with phosphorylated-antibodies, membranes were stripped by reblot plus strong antibody stripping solution according to the manufacturer’s instructions, before re-probing with total antibodies. For quantification, phosphorylated proteins were normalised to their respective total protein; puromycin was normalised to the total protein as determined from the stain-free lane density.

### 4.11. Muscle Protein Synthesis (MPS)

MPS was determined via the surface sensing of translation technique (SUnSET) [42]. Differentiated C2C12 myotubes were nutrient deprived in amino acid- and serum- free DMEM medium for 1 h and subsequently treated with either 1 mg·mL^−1^ protein equivalent hydrolysate, 100 ng·mL^−1^ IGF-1 (positive control), or in amino acid and serum free DMEM (negative control) containing 1 µM puromycin medium containing 1 µM puromycin for a further 4 h. Cellular protein lysates were obtained and MPS was determined by immunoblotting as described above.

### 4.12. Statistical Analysis

The software programme SPSS (Version 27, IBM Inc., Chicago, IL, USA) was used to perform statistical analyses on the data. Data were expressed as mean ± SD for all data. One-way analysis of variance (ANOVA) and *t*-test were used to compare all values. A *p* value < 0.05 was considered as a significant difference.

## Figures and Tables

**Table 1:** The degree of hydrolysis (DH) and molecular mass distribution profiles of the blue whiting (*Micromesistius poutassou*) protein hydrolysates (BWPHs) pre- and post-simulated gastrointestinal digestion (SGID), Data reported as mean ± SD (n = 3). * Indicates a significant difference at *p* < 0.05 between pre- and post-simulated gastrointestinal digestion values.

Test Sample	DH (%)	Molecular Mass Distribution (%)			
		>10 kDa	5–10 kDa	1–5 kDa	<1 kDa
BWPH-X BWPH-X-SGID	25.89 ± 0.21 *	2.13	4.74	15.61	77.5
	37.29 ± 0.24	0	0.25	2.63	97.12
BWPH-Y BWPH-Y-SGID	26.81 ± 0.82 *	1.95	4.97	38.69	54.39
	35.48 ± 0.27	0.01	0.30	13.03	86.67
BWPH-Z BWPH-Z-SGID	27.16 ± 0.68 *	1.51	3.72	32.16	62.6
	33.41 ± 0.91	0.01	0.21	11.04	88.75

**Table 2:** Amino acid (AA) profiles of blue whiting (*Micromesistius poutassou*) protein hydrolysates (BWPHs g·kg^−^^1^).

AA		AA Content of BWPHs (g·kg^−1^)		
		BWPH-X	BWPH-Y	BWPH-Z
Non essential (NEAA)	Cys	5.3	5.4	12.4
	Arg	58.5	58.7	50.1
	Asx	95.2	90.9	82.9
	Pro	37.5	36.7	28.8
	Ser	42.5	40.9	37.1
	Glx	146.0	139.0	133.7
	Gly	62.6	60.2	58.7
	Ala	59.9	57.8	52.6
	Tyr	26.2	25.5	14.6
Essential amino acids (EAA)	Val	31.4	30.1	33.8
	Ile	24.6	23.8	27.3
	Leu	56.2	55.3	53.7
	Trp	5.7	5.3	5.9
	Phe	26.5	26.0	26.3
	His	15.9	15.6	25.3
	Lys	80	75.7	71.5
	Met	19.1	19.2	26.0
	Thr	34.9	34.0	31.5
	EAA	294.3	285.0	301.3
	NEAA	533.7	515.1	470.9
	EAA:NEAA	0.55	0.55	0.6
	BCAA	112.2	109.2	114.9
	TAA	828.0	800.1	772.2

Amino acid residues are denoted by their 3-letter code. Asx: Aspartic acid and asparagine, Glx: Glutamic acid and glutamine. EAAs: essential amino acids. NEAA: non-essential amino acid. BCAA: branched chain amino acid. TAA: total amino acid.

## Data Availability

Data are contained within the article.
